# The Associations between Organophosphate Pesticides (OPs) and Respiratory Disease, Diabetes Mellitus, and Cardiovascular Disease: A Review and Meta-Analysis of Observational Studies

**DOI:** 10.3390/toxics11090741

**Published:** 2023-08-30

**Authors:** Lei Zhao, Qisijing Liu, Yaning Jia, Huishu Lin, Yuanyuan Yu, Xuemei Chen, Ziquan Liu, Weixia Li, Tao Fang, Wenbing Jiang, Jianfeng Zhang, Huanhuan Cui, Penghui Li, Hongyu Li, Shike Hou, Liqiong Guo

**Affiliations:** 1 Institute of Disaster and Emergency Medicine, Tianjin University, Tianjin 300072, China; 2 Tianjin Fourth Central Hospital, Tianjin 300140, China; 3 Wenzhou Safety (Emergency) Institute, Tianjin University, Wenzhou 325000, China; 4 Research Institute of Public Health, School of Medicine, Nankai University, Tianjin 300381, China; 5 The Dingli Clinical College, Wenzhou Medical University, Wenzhou 325000, China; 6 School of Environmental Science and Safety Engineering, Tianjin University of Technology, Tianjin 300384, China; 7 Office for National Clinical Research Center for Geriatric Diseases, Beijing Hospital, Beijing 100051, China; 8 National Center of Gerontology, Institute of Geriatric Medicine, Chinese Academy of Medical Sciences, Beijing 100700, China

**Keywords:** organophosphate pesticides, respiratory diseases, diabetes mellitus, cardiovascular diseases

## Abstract

Although some epidemiological studies have identified the associations between exposure to organophosphate pesticides (Ops) and respiratory diseases, diabetes mellitus (DM), and cardiovascular diseases (CVDs), controversial results still exist. In this review and meta-analysis, we aimed to investigate the overall pooled effect estimates and the possible mechanisms of the relationship between OP exposure and adverse health outcomes. In this study, Web of Science, PubMed, Embase, OVID, and the Cochrane Library were systematically searched until September 2022. Nineteen observational studies that focused on the general population or occupational populations examined the associations between OP exposure and respiratory diseases, DM, and CVD were included. Based on the overall pooled results, a significantly positive association was observed between OP exposure and respiratory diseases (OR: 1.12, 95% CI: 1.06–1.19). A significant link was also observed between various individual species of OP exposure and respiratory diseases, with an OR value of 1.11 (95% CI: 1.05–1.18). In particular, there was a significant association of OPs with wheezing and asthma, with OR values of 1.19 (95% CI: 1.08–1.31) and 1.13 (95% CI: 1.05–1.22), respectively. In addition, a significant association was also observed between OP exposure and DM (OR: 1.18, 95% CI: 1.07–1.29). However, no significant association was observed between OP exposure and CVD (OR: 1.00, 95% CI: 0.94–1.05). Exposure to OPs was associated with a significantly increased risk of respiratory diseases and DM, but there was no evidence of a significant association between OP exposure and CVD. Considering the moderate strength of the results, further evidence is needed to confirm these associations.

## 1. Introduction

Organophosphate pesticides (OPs) are a common kind of pesticide that is widely used for agriculture, livestock, and other commercial purposes around the world [1]. Since organochlorine (OC) pesticides have been banned in most countries for decades [2], OPs became a major contributor to the pesticide market in the United States in 2012, accounting for 30% of all pesticides [3]. The wide usage of OPs has resulting in their being easily accumulated in the environment, especially in the air [4], water [5], soil, and food resources [6] (Presence and Health Risks of Obsolete and Emerging Pesticides in Paddy Rice and Soil from Thailand and China), and the residues of OPs can gradually increase the risk of occupational and accidental exposure. Studies have proved that exposure to OPs has a great effect on the health of humans and animals, as they can induce neurotoxicity, genotoxicity, cardiotoxicity, immunotoxicity, hepatotoxicity, nephrotoxicity, reproductive toxicity, and metabolic diseases [7]. The toxicity of OPs can induce an imbalance of oxidant and antioxidant components in neuron cells [8]. In addition, most OPs have potent pro-oxidant activity, which can cause disturbances in the mitochondrial function in neurons, leading to various neurological diseases [9].

Indeed, several previous studies have noted that exposure to pesticides has an effect on many adverse health outcomes. In a meta-analysis conducted by Ratanachina et al. [10], the pooled results of 56 articles showed that exposure to cholinesterase (ChE)-inhibiting pesticides could induce a decrement in forced expiratory volume in 1 s (FEV_1_)/forced vital capacity (FVC), which is an important indicator of lung function. Evangelou et al. revealed an elevated diabetes risk associated with exposure to organochlorine pesticides, including dichlorodiphenyldichloroethylene (DDE), heptachlor, hexachlorobenzene (HCB), dichlorodiphenyltrichloroethane (DDT), and trans-nonachlor or chlordane [11]. In addition, it was reported that environmental contamination, such as contamination with tetrachlorodibenzo-p-dioxin, organochlorine, and heavy metals, was associated with CVD risk in a meta-analysis conducted by Zago et al. [12]. However, these studies were focused on various kinds of pesticides, which potentially induced a bias when assessing the individual pesticides’ effects. Thus, due to their toxicity and wide use in common life, it is necessary to present a summary of evidence of the associations between OP exposure and adverse health outcomes. In recent years, many observational studies have indicated that OP exposure can increase the risk of respiratory disease, diabetes mellitus (DM) [13], and cardiovascular disease (CVD) [14,15,16]. Through the primary exposure routes of OPs—inhalation, ingestion of contaminated food, and dermal contact [17]—they can covalently and irreversibly bind with acetylcholinesterase (AChE) at its active site and then inhibit the activity of AChE, which may impair the airway and the lung functions [18]. In addition, AChE inhibition also increases the accumulation of acetylcholine, which can reduce insulin secretion; thus, insulin is not adequate to stimulate the uptake of adequate glucose in adipose and muscle tissues, leading to hyperglycemia and DM [13]. OPs also have an impact on the human central nervous system, and central nervous system toxicity may be a common route for CVD development by leading to the generation of oxidative stress in the cardiovascular system and resulting in alterations of proteins, such as through accelerated degradation [16]. Evidence from human and animal studies supported the potential role of OPs in the development of three diseases: respiratory disease, DM, and CVD [14,19,20,21,22,23].

However, several epidemiological studies have reported inconsistent results regarding associations between OP exposure and respiratory disease, DM, and CVD. No evidence of respiratory dysfunction and asthma after chronic OP exposure was found in two occupational cohorts and a cohort of children [24,25,26]. Similarly, there was insufficient evidence to link OP exposure with plasma glucose and insulin resistance in the general populations of the US and Thailand [27,28]. In addition, the effects of OP-induced CVD were also inconclusive [29]. Given that the results were inconsistent regarding OP exposure and the development of respiratory disease, DM, and CVD, here, we systematically examined this topic. A review and meta-analysis of available research were needed to investigate the underlying impacts of OP exposure on the incidence of respiratory disease, DM, and CVD. Thus, we designed this review and meta-analysis of observational studies on the associations between OP exposure and respiratory disease, DM, and CVD to summarize the current findings (until September 2022).

## 2. Methods

### 2.1. Search Strategy for the Studies Included

This study was conducted based on the Preferred Reporting Items for Systematic Reviews and Meta-Analyses (PRISMA) guide [30]. Web of Science, PubMed, Embase, Cochrane Library, and OVID were comprehensively searched until the date of 2 September 2022 to identify studies that evaluated the associations between OP exposure and respiratory disease, DM, and CVD. The keywords related to exposure used in the databases were “organophosphorus pesticide”, “organophosphorus insecticide”, or “OPs”, and the keywords related to health outcomes were “respiratory disease”, “lung disease”, “pulmonary disease”, “diabetes mellitus”, “DM”, “T2DM”, “diabetes”, “cardiovascular disease”, “heart disease”, “kidney disease”, “kidney failure”, “renal disease”, “renal failure”, “renal insufficiency”, “liver disease”, “liver function”, or “liver fibrosis”. We also checked the reference lists of related studies to avoid missing any relevant research. The details of the search strategies employed in this study are presented in Supplementary File S1.

### 2.2. Criteria for Study Selection

The included studies were all in agreement with the principle of PICOS (population, intervention, control, outcomes). The following specific criteria were considered as the inclusion criteria in this meta-analysis: (1) The study design of eligible studies should be that of a cross-sectional study, cohort study, or case–control study. (2) The study subject was the general population or occupational populations. (3) The studies involved OP exposure (mixed or individual). (4) The health outcomes of the population were respiratory disease, DM, or CVD. (5) The risk estimates between OP exposure and respiratory disease, DM, or CVD reported in the eligible studies needed to be presented as the odds ratio (OR), relative risk (RR), or hazard ratio (HR) with 95% confidence intervals (95% CIs). We excluded the studies if they (1) were review articles, editorials, commentaries, conference proceedings, or case reports, (2) were non-epidemiological studies, such as in vitro or in vivo animal studies, (3) did not examine the field of interest, or (4) were written in a language other than English.

### 2.3. Data Extraction

Two authors (L.Z. and Q.L.) performed the evaluation of all records and then independently extracted the data. The initial evaluation was conducted by screening the titles, abstracts, and keywords based on the criteria for inclusion and exclusion. EndNote (X9), a reference manager software, was employed to delete the duplicated research and excluded studies. Full-text browsing screening was performed by the same two authors to obtain the potentially eligible studies. Another author (L.G.) was consulted when disagreements were encountered.

The data extracted from the eligible studies included the following: the name of the first author, publication year, study design, study location, sample size, ethnicity of the participants (people who lived on different continents, such as Asia and the Americas), characteristics of the participants (age, gender), types of OPs, disease outcomes, effect estimates (OR, RR, or HR with 95% CIs), and confounding factors from each study. If a comprehensive cohort was investigated in different studies, only the study containing the latest data was included in this meta-analysis.

### 2.4. Quality Assessment

A quality assessment of the studies included in this meta-analysis was conducted by two independent authors (L.Z. and Q.L.), and any controversies were addressed through comprehensive discussions. Quality was assessed by using the nine-star Newcastle–Ottawa scale (NOS) [31], which consists of three sections—participant selection, comparability, and outcomes of interest. The scores of the NOS range from 0 to 9; 0–5 stars were considered as low quality, and 6–9 stars indicated high quality.

### 2.5. Search Strategy for the Included Studies

The values of the OR and 95% CI were considered as the effect size and were extracted from all included studies. The pooled ORs with 95% CIs for respiratory diseases, DM, and CVD were calculated by using a random-effect model to minimize the heterogeneity between different studies. The heterogeneity between studies was quantified by using the Chi-square-based Cochrane Q statistic test and the standard *I*^2^ statistic; *I*^2^ > 50 was considered as indicating medium heterogeneity and *I*^2^ > 75% was considered as indicating high heterogeneity. The results of this meta-analysis were displayed by using forest plots based on the year of publication of the studies. We conducted a subgroup analysis to examine the possible sources of heterogeneity; the subgroups were stratified by disease, ethnicity, individual OPs, and study design.

In addition, to obtain the pooled results for various types of OPs, the effect sizes of few individual OPs in every included study were combined to calculate the final results [32]. Publication bias was examined by using funnel plots and the results of Egger’s test, and the significance level in this study was set to *p* < 0.05 in Egger’s test. The sensitivity analyses were performed by removing one study at a time to evaluate whether the significance of the results was altered if any studies were omitted. The entire process of statistical analysis was conducted by using the STATA software, version 16.0 (StataCorp., College Station, TX, USA).

## 3. Results

### 3.1. Study Selection

**Figure 1:**
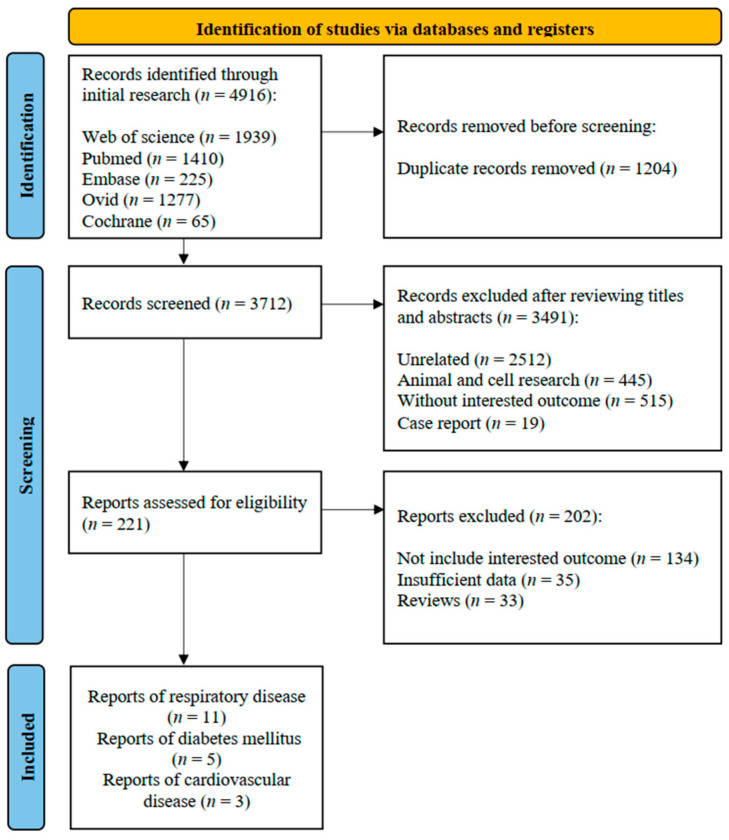
PRISMA flow diagram of the literature search in this study.

The PRISMA flow diagram for study selection in this meta-analysis is presented in Figure 1. After the initial search, 4916 publications were obtained from five main databases, namely, Web of Science (*n* = 1939), Pubmed (*n* = 1410), Embase (*n* = 225), OVID (*n* = 1277), and the Cochrane Library (*n* = 65). After removing the duplicates, 3712 articles were retained for title and abstract screening. In accordance with the inclusion and exclusion criteria, 3491 studies were excluded, and 221 studies were accessed for a full-text evaluation. Of these studies, 134 studies did not report the outcome of interest, 35 studies lacked sufficient data, and 33 studies were reviews. One study [33] investigated the same cohort from the same population (farmers and commercial pesticide applicators) as that used in two other studies [34,35]. Accordingly, only the studies containing the most recent data for farmers [33] and commercial applicators of pesticides [35] were included in the meta-analysis. Finally, 19 studies were eligible for data extraction and further analysis in this meta-analysis. Among these studies, 11 investigated the association between OP exposure and respiratory disease, 5 investigated OPs and DM, and 3 investigated OPs and CVD.

### 3.2. Study Characteristics

The overall characteristics of the 19 included studies are presented in Table 1. These articles were published from 2006 [33] to 2020 [3,36] and were conducted in the USA [3,13,33,35,37,38,39,40,41], India [42,43], China [15,44], Chile [45], Thailand [46], Sri Lanka [36], and Costa Rica [22]. The sample size of these studies ranged from 127 [22] to 46,115 [44], and the age range of the participants was from 6 to 88. Among them, 11 studies examined respiratory disease, 5 studies investigated the association between OP exposure and asthma [3,22,37,38,45], 3 studies investigated wheezing [33,35,38], 2 studies investigated chronic obstructive pulmonary disease (COPD) [39,44], one study investigated chronic bronchitis [3], and other studies did not specify the respiratory diseases [36,42]. Three studies reported the relationship between CVD and OP exposure, one study reported the link between hypertension and OPs [45], one study investigated coronary artery disease [15], and the last one did not specify the kind of CVD [41]. In addition, five studies reported the association between OP exposure and DM [13,40,43,45,46]. Most of the studies were conducted as cohort studies (11 studies), 7 studies were designed as cross-sectional studies, and one study was case-control study. Four studies investigated various diseases in the same population [3,15,22,45]; among these four studies, Sun et al. stratified the whole population into different subgroups according to age and gender, and they examined the association between OP exposure in the subgroups and various diseases [3]. Eleven studies reported individual OP exposure, the other studies evaluated mixed OP exposure.

### 3.3. The Association between OP Exposure and Respiratory Diseases

**Figure 2:**
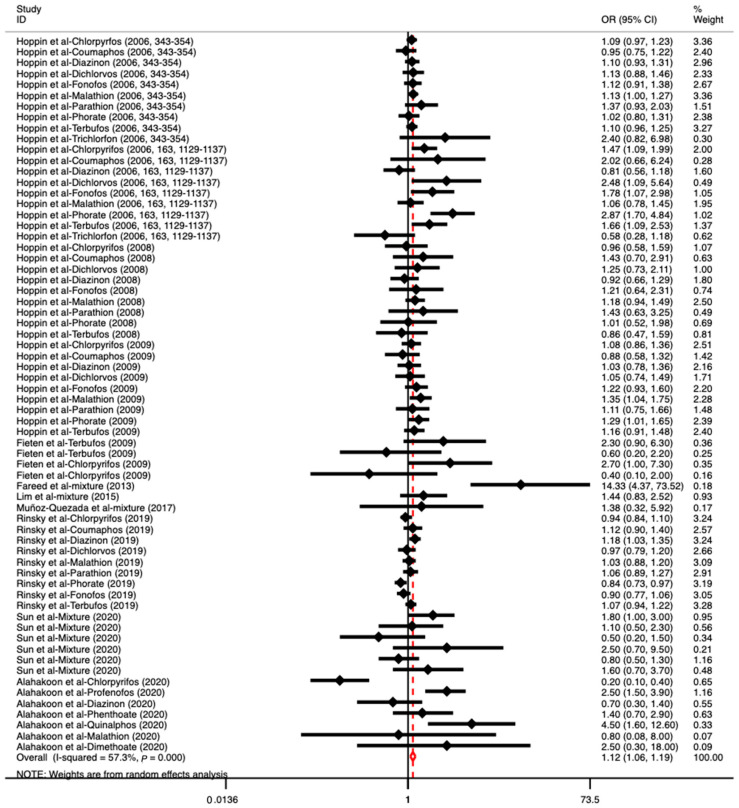
The overall associations between OP exposure (individual and mixed) and respiratory diseases (wheezing, asthma, and other respiratory diseases) ([3,22,33,35,36,37,38,39,42,44,45]). Note: Seven studies reported several effect sizes based on various types of OPs, and only one study reported the associations between mixed OP exposure and respiratory diseases in different age ranges and gender.

In this meta-analysis, 11 studies that examined the associations between individual or mixed OP exposure and different respiratory diseases were found. All included studies received acceptable quality scores that ranged from 6 to 8 based on the NOS, except for one study [36], which had a score of 5 and was considered to have a low quality. However, due to the limited number of studies, that study [36] was also included in the subsequent analysis. The overall pooled OR value using the random-effect model showed a significant positive association between OP exposure and respiratory diseases (OR: 1.12, 95% CI: 1.06–1.19) (Figure 2), and the results of the heterogeneity test suggested that there was an acceptable level of heterogeneity (I2 = 57.30%, *p* < 0.0001). A visual check of the funnel plot and the results of Egger’s linear regression test indicated some evidence of publication bias (*p* = 0.022) (Figure S1, Supplementary File S2).

**Figure 3:**
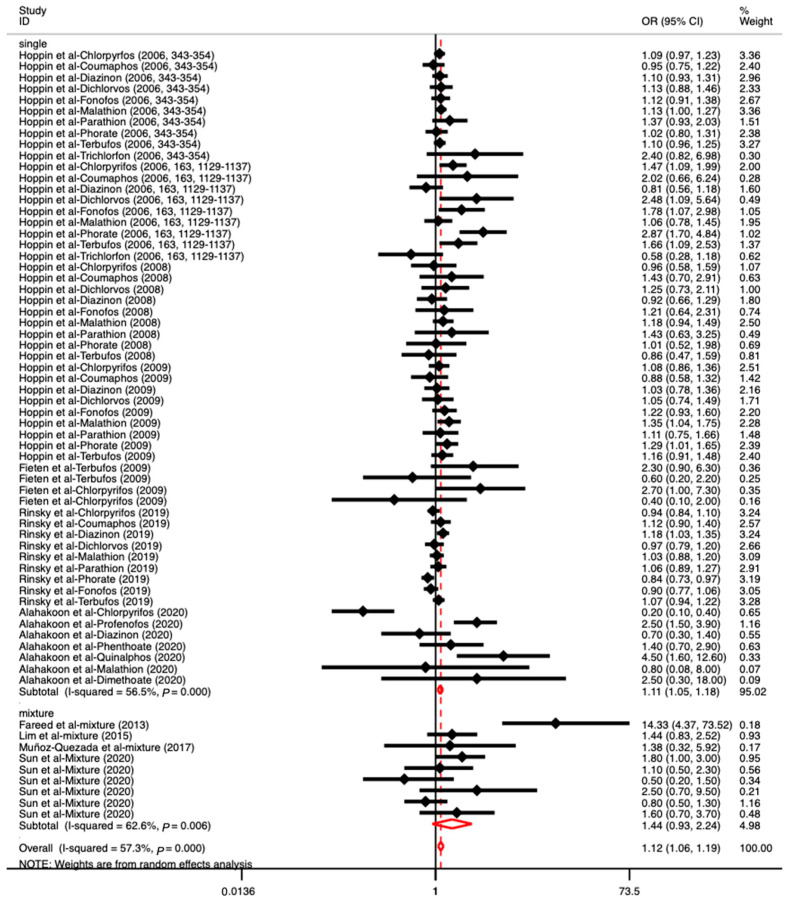
The associations between exposure to different types of OPs (individual and mixed) and respiratory diseases ([3,22,33,35,36,37,38,39,42,44,45]). Note: Seven studies reported several effect sizes based on various types of OPs.

**Figure 4:**
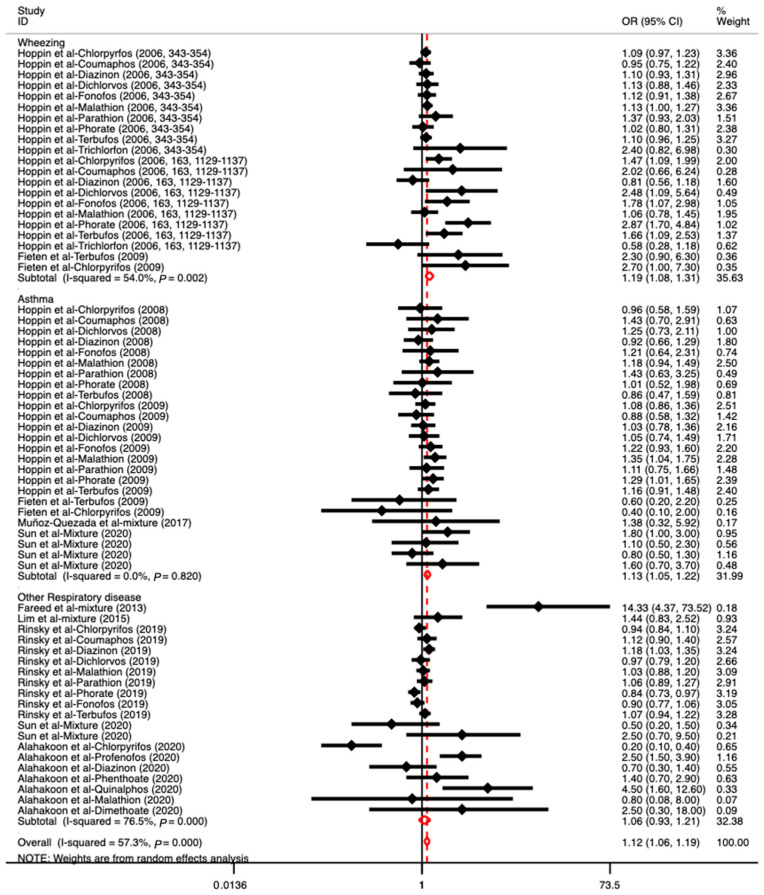
The associations between OP exposure and different respiratory diseases (wheezing, asthma, and other respiratory diseases) ([3,22,33,35,36,37,38,39,42,44,45]). Note: Three studies reported an association between OPs and wheezing, five studies reported an association between OPs and asthma, and five studies reported an association between OPs and other respiratory diseases.

A subgroup analysis was conducted according to the types of OPs (individual or mixed) to which the subjects were exposed. As Figure 3 shows, the results indicated that individual OP exposure had a significant association with respiratory diseases, with an OR value of 1.11 (95% CI: 1.05–1.18), especially for malathion and terbufos (OR: 1.05, 95% CI: 1.02–1.09; OR: 1.05, 95% CI: 1.01–1.09). More details are presented in Figure S2. However, other individual OPs did not show significant associations with respiratory diseases. In addition, no significant associations were observed between mixed OP exposure and respiratory diseases (OR: 1.44, 95% CI: 0.93–2.24). We conducted another subgroup analysis based on the type of respiratory disease. Due to the limited numbers of included articles for a certain respiratory disease (less than three), we summarized these studies as dealing with other respiratory diseases. As Figure 4 shows, there was a significant association of OPs with wheezing and asthma, with OR values of 1.19 (95% CI: 1.08–1.31) and 1.13 (95% CI: 1.05–1.22), respectively. No significant associations between OPs and other respiratory diseases were found (OR: 1.06, 95% CI: 0.93–1.21). We also performed another subgroup analysis to find more results based on the participants’ ethnicity and gender and the design of the studies; for instance, it can be determined which study design was more sensitive and appropriate for investigating the associations between OP exposure and respiratory diseases (Figures S3–S5, Supplementary File S2). The results showed that American ethnicity (North American and South American), male, and cohort study design exhibited a positive association between OPs and respiratory diseases, with ORs of 1.10 (95% CI: 1.05–1.16), 1.16 (95% CI: 1.01–1.34), and 1.11 (95% CI: 1.05–1.18), respectively.

### 3.4. The Association between OP Exposure and DM

**Figure 5:**
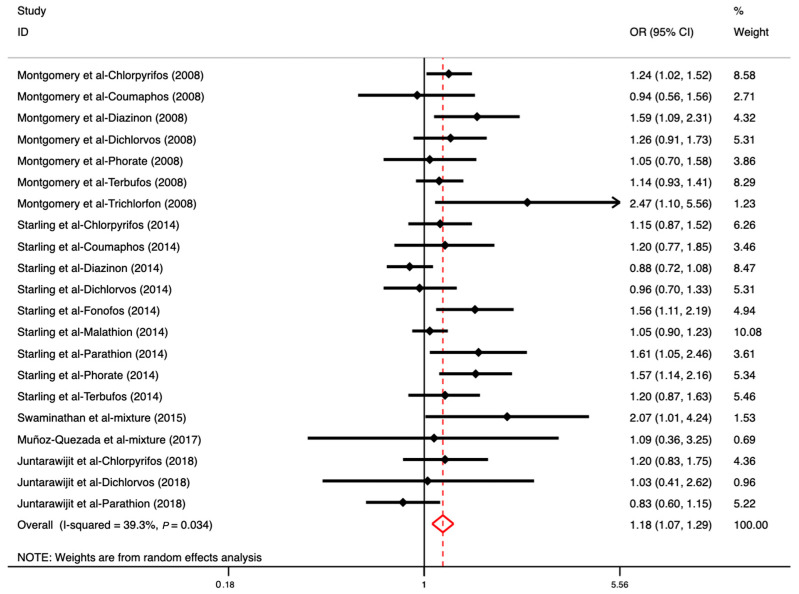
The overall association between OP exposure (individual and mixed) and DM ([13,40,43,45,46]). Note: Three studies reported several effect sizes based on various types of OPs.

Five of the included studies evaluated the association between OP exposure and DM incidence. After all results were pooled with the random-effect model, a significant association between OPs and DM was identified (OR: 1.18, 95% CI: 1.07–1.29), and the heterogeneity level was acceptable (*I^2^* = 39.30%, *p* < 0.05) (Figure 5). The funnel plot and Egger’s linear regression test are presented in Figure S6 in Supplementary File S2; they indicated that there was no evidence of publication bias (*p* = 0.073). Due to the limited number of studies included, the subgroup analysis considered only the study design as a factor. As Figure S7 in Supplementary File S2 shows, cohort studies indicated a significant association between OPs and DM (OR: 1.19, 95% CI: 1.08–1.32).

### 3.5. The Association between OP Exposure and CVD

**Figure 6:**
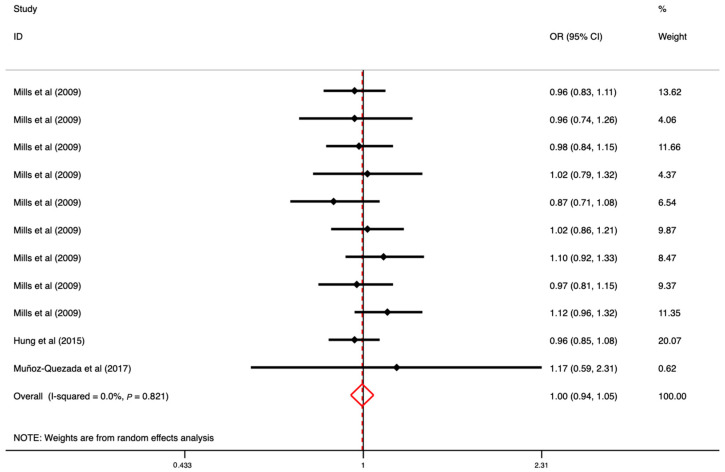
The overall associations between OP exposure (individual and mixed) and CVD ([15,41,45]). Note: One study reported several effect sizes based on various types of OPs, and one study reported various CVDs, including arrhythmia, coronary artery disease, and congestive heart failure.

Three studies examined the association of OPs with CVD incidence; the overall pooled results showed that there were no significant associations between OPs and CVD by using the random-effect model (OR: 1.00, 95% CI: 0.94–1.05) (*I*^2^ = 0.0%, *p* = 0.821) (Figure 6). The results of the funnel plot and Egger’s linear regression test indicated that there was no publication bias between OPs and CVD (*p* = 0.64) (Figure S8, Supplementary File S2). Since a limited number of studies reported the association of OPs with CVD, no subgroup analysis was performed in the further analysis.

## 4. Discussion

In this meta-analysis, we have summarized the evidence for exposure to various OPs and its associations with respiratory diseases, DM, and CVD. The nineteen epidemiological articles included in the current study were mostly cohort and cross-sectional studies. The findings indicated a positive association between OP exposure and respiratory diseases, and the subgroup analysis also showed that the type of OP, specific respiratory disease, ethnicity, gender, and study design were important factors in the association between OP exposure and respiratory diseases. In addition, a significant association between OPs and DM was also observed by pooling five studies, and the subgroup analysis highlighted that study design may play an important role in the association between OP exposure and DM. On the other hand, our results did not show any substantial relationship between OP exposure and CVD incidence, which might be due to the limited number of studies included. These findings suggest the potential threat to human health due to exposure to OPs, especially in terms of respiratory diseases and DM.

A great number of epidemiological studies provided evidence showing that OP exposure was associated with wheezing and asthma [47,48,49,50], which was consistent with the results of our subgroup analysis. The major pathophysiological pathways through which OP exposure might promote the risk of respiratory diseases include the inhibition of AChE and accumulation of Ach, which might induce overstimulation throughout the central and peripheral nervous systems [51]. The exact mechanisms of the effect of OP exposure on the peripheral nervous system include hypotension (via muscarinic and non-muscarinic mechanisms) [52,53], weakness and paralysis caused by effects on the neuromuscular junction [54], and bradycardia, bronchoconstriction, and bronchorrea caused by the effects of muscarinic effects. The central effects induced by OP exposure include central respiratory depression, but the mechanisms are still unclear.

Furthermore, the subgroup analyses indicated that OP exposure might be linked with a higher risk of respiratory diseases in American populations. This is likely due to the overwhelming number of studies conducted in America; eight out of the eleven included studies were conducted in North or South America, which might have introduced some bias into this meta-analysis. In the current study, a significant association between OP exposure and respiratory diseases was observed in males, which might be because the study participants were mostly selected from occupational cohorts, which are dominated by males.

Previous studies have also suggested several biological mechanisms for the link between OPs and DM. Indeed, according to some animal research, chronic exposure to OPs could cause an increment in the body weight of rats when compared with controls [55]. Lassiter et al. found similar results—OP exposure was able to cause excessive weight gain and impaired production of leptin in male rats [56]. Another study proved that OP exposure could disrupt the homeostasis of fat and glucose; one possible pathway was the adenylyl cyclase/cyclic AMP pathway, which plays an important role in the increased risk of obesity and diabetes [57]. In addition, growing evidence from epidemiological studies has shown that OP exposure could mediate damage to pancreatic β cells, insulin resistance, and excessive hepatic gluconeogenesis, which are all possible contributors to the development of DM. A study conducted by Panda et al. proved that OP exposure in the general population has a potential link with a higher level of insulin resistance and plasma glycated hemoglobin [58]. A clinical study reported that, in an OP-exposed group, the activity of AChE was decreased in red blood cells, whereas the concentration of lipase/amylase and insulin in plasma was increased, demonstrating direct damage to the pancreatic cells due to OP exposure [59]. In addition, after OP poisoning, the levels of malondialdehyde (MDA) and superoxide dismutase were elevated and reduced glutathione was depleted, indicating the generation of reactive oxygen species (ROS) [60]. ROS could directly interfere with the signaling of insulin receptors by activating the serine residues on insulin receptor substrate 1 and then inhibiting glucose-transported type 4 [61]. Thus, the ROS generated by OPs could also mediate insulin resistance.

Moreover, recent studies have also suggested an association between OP exposure and CVD risk factors. In particular, Allon et al. conducted a study to examine the effects of AChE inhibitors on arrhythmias in rats, and the results showed that excessive Ach after OP poisoning could be a potential etiological factor for arrhythmias [62]. In addition, OPs could cause the inhibition of AChE and paraoxonase (PON1) consumption through two pathways [15]. First, OPs combined with plasma AChE; second, cytochrome P450 bioactivates OPs to create highly toxic oxon forms, and they are then hydrolyzed by PON1 into harmless products [63]. Another study conducted by Xiong et al. proved that PON1 could protect vessel walls against damage through antioxidation and elimination of oxidation products [64]. Thus, the decrement in PON1 caused by OP exposure might be implicated in the pathogenesis of CVD [65]. Actually, studies focused on the impacts of OP exposure on adverse health outcomes were mostly conducted in occupational populations, so future studies need to concentrate on the general population as well. Our meta-analysis has several strengths; the current study is the first review and meta-analysis of the associations between OP exposure and the risk of respiratory diseases, DM, and CVD in general or occupational populations. Subgroup and sensitivity analyses were also performed in this meta-analysis. However, there were some limitations in our study that need to be noted. For instance, due to the limited number of studies about single outcomes and OP exposure, we reported the pooled results for respiratory diseases and CVD, which is a common and reliable method used in meta-analyses [32]. However, the accuracy of the pooled results may be lower than that of those concerning a single, well-defined health outcome. In addition, some confounders in the studies that were included in this meta-analysis were not adjusted, and bias might have been introduced by pooling the same results from one study several times. The subgroup analyses between OP exposure and respiratory diseases, especially for study design, were not accurate enough due to the limited number of cross-sectional studies included. In addition, this meta-analysis included individuals from occupational populations; thus, the pooled results for adverse health outcomes may have been amplified due to long-term exposure to OPs. Moreover, due to the limited number of studies that investigated the relationship between OP exposure and CVD, the result of the pooled effect size in this study was not accurate enough, and a subgroup analysis was not performed. There is a need for more evidence and data on the relationship between OPs and the risk of CVD; future studies should be focused on that.

## 5. Conclusions

This review and meta-analysis included 19 studies and suggests a relationship of OP exposure with respiratory diseases and diabetes mellitus, with overall pooled OR values of 1.12 (95% CI: 1.06–1.19) and 1.18 (95% CI: 1.07–1.29), respectively. Further study is required to investigate the relationships between OPs and adverse health outcomes in general populations in order to obtain more accurate results, and more confounders should be considered to adjust the final results.

## Figures and Tables

**Table 1:** The characteristics of the studies that were included.

Author (Year)	Country	Age (Range or Mean ± SD)	Sample Size	Study Design	Outcome	Exposure	Comparison	OR (95% CI)	Adjustment	Quality Score
Hoppin et al., 2006 [33]	USA	18–88	17,920 (F: 538, M: 17,382)	Cohort	Respiratory disease (wheezing)	Chlorpyrifos Coumaphos Diazinon Dichlorvos Fonofos Malathion Parathion Phorate Terbufos Trichlorfon	Population who never reported using OPs	1.09 (0.97–1.23) 0.95 (0.75–1.22 1.10 (0.93–1.31) 1.13 (0.88–1.46) 1.12 (0.91–1.38) 1.13 (1.00–1.27) 1.37 (0.93–2.03) 1.02 (0.80–1.31) 1.10 (0.96–1.25) 2.40 (0.82–6.98)	Age, BMI, smoking, asthma/atopy status, and previous use of pesticide	7
Hoppin et al., 2006 [35]	USA	17–83	2255 (F: 114, M: 2141)	Cohort	Respiratory disease (wheezing)	Chlorpyrifos Coumaphos Diazinon Dichlorvos Fonofos Malathion Phorate Terbufos Trichlorfon	The group “Never use” was the reference category	1.47 (1.09–1.99) 2.02 (0.66–6.24) 0.81 (0.56–1.18) 2.48 (1.09–5.64) 1.78 (1.07–2.98) 1.06 (0.78–1.45) 2.87 (1.70–4.84) 1.66 (1.09–2.53) 0.58 (0.28–1.18)	Age, BMI, smoking status, asthma/atopy status	7
Hoppin et al., 2008 [37]	USA	20–88	25,814 (F)	Cohort	Respiratory disease (asthma)	Chlorpyrifos Coumaphos Dichlorvos Diazinon Fonofos Malathion Parathion Phorate Terbufos	Nonexposed population as the reference	0.96 (0.58–1.59) 1.43 (0.70–2.91) 1.25 (0.73–2.11) 0.92 (0.66–1.29) 1.21 (0.64–2.31) 1.18 (0.94–1.49) 1.43 (0.63–3.25) 1.01 (0.52–1.98) 0.86 (0.47–1.59)	Age, state, BMI, smoking status, and “grew up on farm”	7
Hoppin et al., 2009 [38]	USA	≥20	19,704 (M)	Cohort	Respiratory disease (asthma)	Chlorpyrifos Coumaphos Diazinon Dichlorvos Fonofos Malathion Parathion Phorate Terbufos	Never-users were the reference group	1.08 (0.86–1.36) 0.88 (0.58–1.32) 1.03 (0.78–1.36) 1.05 (0.74–1.49) 1.22 (0.93–1.60) 1.35 (1.04–1.75) 1.11 (0.75–1.66) 1.29 (1.01–1.65) 1.16 (0.91–1.48)	Age, state, BMI, smoking, high pesticide exposure events	7
Fieten et al., 2009 [22]	Costa Rica	20–58	127 (M)	Cross-sectional study	Respiratory disease (wheezing)	Chlorpyrifos Terbufos	Unexposed participants who worked on organic banana plantations or at other locations (home, school, etc.)	2.70 (1.00–7.30) 2.30 (0.90–6.30)	Age and atopic symptoms, defined as self-reported symptoms of rhinitis, eczema, or both, during the last year.	7
					Respiratory disease (asthma)	Chlorpyrifos Terbufos		0.40 (0.10–2.00) 0.60 (0.20–2.20)		
Fareed et al., 2013 [42]	India	38.12 ± 15.39	243 (M)	Cross-sectional study	Respiratory disease	Mixed OPs (such as monocrotphos, dichlorvos, malathion, parathion)	Participants who did not handle pesticides and had a similar socioeconomic status and age group to the exposed subjects	14.33 (4.37–73.52)	Smoking habits	6
Lim et al., 2015 [44]	China	53.40 ± 16.50	46,115 (F: 13,810, M: 32,305)	Cohort	Respiratory disease (COPD)	Mixed OPs	Population without OP poisoning	1.44 (0.83–2.52)	Age and comorbidities of atrial fibrillation, hypertension, diabetes, CVA, and heart failure.	8
Muñoz-Quezada et al., 2017 [45]	Chile	49.00 ± 12.60	207 (F: 102, M: 105)	Cross-sectional study	Respiratory disease (asthma)	Mixed OPs	Non-agricultural workers (non-exposed)	1.38 (0.32–5.92)	-	7
Rinsky et al., 2019 [39]	USA	27–97	22,491 (F: 621, M: 21,870)	Cohort	Respiratory disease (COPD)	Chlorpyrifos Coumapphos Diazinon Dichlorovs Malathion Parathion Phorate Fonofos Terbufos	Farmers who did not report a diagnosis or symptoms consistent with chronic bronchitis	0.94 (0.84–1.10) 1.12 (0.90–1.40) 1.18 (1.03–1.35) 0.97 (0.79–1.20) 1.03 (0.88–1.20) 1.06 (0.89–1.27) 0.84 (0.73–0.97) 0.90 (0.77–1.06) 1.07 (0.94–1.22)	Using stabilized inverse probability of exposure weights (IPEWs) to address confounding factors	6
Sun et al., 2020 [3]	USA	6–19	1830 (F)	Cross-sectional study	Respiratory disease (asthma)	Mixed OPs	Participants who had the lowest OP metabolite levels	1.80 (1.00–3.00)	BMI, creatinine, and races	7
		20–39	1181 (F)		Respiratory disease (asthma)			1.10 (0.50–2.30)		
		40–59	1036 (F)		Respiratory disease (chronic bronchitis)			0.50 (0.20–1.50)		
		60–85	1056 (F)		Respiratory disease (chronic bronchitis)			2.50 (0.70–9.50)		
		6–19	1794 (M)		Respiratory disease (asthma)			0.80 (0.50–1.30)		
		20–39	1079 (M)		Respiratory disease (asthma)			1.60 (0.70–3.70)		
Alahakoon et al., 2020 [36]	Sri Lanka	25–49	540 (F: 166, M: 374)	Cohort	Respiratory disease	Chlorpyrifos Profenofos Diazinon Phenthoate Quinalphos Malathion Dimethoate	Comparing the odds of one OP with the odds for all other confirmed OPs combined	0.20 (0.10–0.40) 2.50 (1.50–3.90) 0.70 (0.30–1.40) 1.40 (0.70–2.90) 4.50 (1.60–12.60) 0.80 (0.08–8.00) 2.50 (0.30–18.00)	-	5
Montgomery et al., 2008 [13]	USA	<40, 40–49, 50–59, 60–69, ≥70	37,787 (F: 832, M: 30,955)	Cohort	DM	Chlorpyrifos Coumaphos Diazinon Dichlorvos Phorate Terbufos Trichlorfon	Participants who never used OPs were the reference category	1.24 (1.02–1.52) 0.94 (0.56–1.56) 1.59 (1.09–2.31) 1.26 (0.91–1.73) 1.05 (0.70–1.58) 1.14 (0.93–1.41) 2.47 (1.10–5.56)	Age, BMI, and state	7
Starling et al., 2014 [40]	USA	17–88	13,637 (F)	Cohort	DM	Chlorpyrifos Coumaphos Diazinon Dichlorvos Fonofos Malathion Parathion Phorate Terbufos	Participants who reported no diabetes	1.15 (0.87–1.52) 1.20 (0.77–1.85) 0.88 (0.72–1.08) 0.96 (0.70–1.33) 1.56 (1.11–2.19) 1.05 (0.90–1.23) 1.61 (1.05–2.46) 1.57 (1.14–2.16) 1.20 (0.87–1.63)	BMI and state at enrollment	7
Swaminathan et al., 2015 [43]	India	>18	260 (F and M)	Cross-sectional study	DM	Mixed OPs	No or minimal exposure group (participants working in offices or people at home)	2.07 (1.01–4.24)	-	5
Muñoz-Quezada et al., 2017 [45]	Chile	49.00 ± 12.60	207 (F: 102, M: 105)	Cross-sectional study	DM	Mixed OPs	Non-agricultural workers (non-exposed)	1.09 (0.36–3.25)	-	7
Juntarawijit et al., 2018 [46]	Thailand	15–86	1,887 (F: 1244, M: 643)	Case-control study	DM	Chlorpyrifos Dichlorvos Parathion	For each specific pesticide, exposure was categorized as ever vs. never used.	1.20 (0.83–1.75) 1.03 (0.41–2.62) 0.83 (0.60–1.15)	Age, gender, BMI, smoking status, alcohol consumption, family history of diabetes, and occupation	8
Mills et al., 2009 [41]	USA	<50, 50–59, 60–69, >69	32,024 (M)	Cohort	CVD	Chlorpyrifos Coumaphos Diazinon Dichlorvos Fonofos Malathion Parathion Phorate Terbufos	Participants with no exposure to each individual pesticide	0.96 (0.83–1.11) 0.96 (0.74–1.26) 0.98 (0.84–1.15) 1.02 (0.79–1.32) 0.87 (0.71–1.08) 1.02 (0.86–1.21) 1.10 (0.92–1.33) 0.97 (0.81–1.15) 1.12 (0.96–1.32)	Age, BMI, state, and smoking status	7
Hung et al., 2015 [15]	China	39.0–63.6	37,805 (F: 11,010, M: 26,795)	Cohort	CVD (coronary artery disease)	Mixed OPs	Population without OP poisoning	0.96 (0.85–1.08)	Age, gender, and comorbidities of diabetes, hypertension, hyperlipidemia, and COPD	7
Muñoz-Quezada et al., 2017 [45]	Chile	49.00 ± 12.60	207 (F: 102, M: 105)	Cross-sectional study	CVD (hypertension)	Mixed OPs	Non-agricultural workers (non-exposed)	1.17 (0.59–2.31)	-	7

## Data Availability

The data used to support the findings of this study are included within the article and the supplementary information file(s) or are available from the corresponding author upon request.

## Supplementary Materials

The following supporting information can be downloaded at: https://www.mdpi.com/article/10.3390/toxics11090741/s1, Supplementary File S1: Search strategy; Supplementary File S2: Figures of contents: Figure S1: Funnel plot for the association between organophosphate pesticides and respiratory diseases; Figure S2: The significant association between individual organophosphate pesticide exposure and respiratory diseases; Figure S3: The association between organophosphate pesticide exposure and participants’ ethnicity (American and Asian) in the respiratory disease group; Figure S4: The association between organophosphate pesticide exposure and participants’ gender in the respiratory disease group; Figure S5: The association between organophosphate pesticide exposure and study design in the respiratory diseases group; Figure S6: Funnel plot for the association between organophosphate pesticides and diabetes mellitus; Figure S7: The association between organophosphate pesticide exposure and study design in the diabetes mellitus group; Figure S8: Funnel plot for the association between organophosphate pesticides and cardiovascular disease. Reference [66] are cited in the supplementary materials.
