# Supplementary material for: The Associations between Organophosphate Pesticides (OPs) and Respiratory Disease, Diabetes Mellitus, and Cardiovascular Disease: A Review and Meta-Analysis of Observational Studies
Source: Toxics. 2023 Aug 30;11(9):741. doi: 10.3390/toxics11090741 (PMC10535340; doi:10.3390/toxics11090741)
Supplement: Supplementary file 1 [file toxics-11-00741-s001.zip › Supplementary File S1.pdf]

Supplementayr material——Search strategy

Search strategy: date-2nd September 2022

Pubmed: ((diabetes mellitus) OR (DM) OR (T2DM) OR (diabetes) OR (respiratory disease) OR (pulmonary disease) OR (lung disease) OR (cardiovascular disease) OR (heart disease) OR (kidney disease) OR (kidney failure) OR (renal disease) OR (renal failure) OR (renal insufficiency) OR (liver disease) OR (liver function) OR (liver fibrosis)) AND ((organophosphorus pesticide) OR (OPs) OR (organophosphorus insecticide))
1410

Web of science: (TS=(diabetes mellitus) OR TS=(DM) OR TS=(T2DM) OR TS=(diabetes) OR TS=(respiratory disease) OR TS=(pulmonary disease) OR TS=(lung disease) OR TS=(cardiovascular disease) OR TS=(heart disease) OR TS=(kidney disease) OR TS=(kidney failure) OR TS=(renal disease) OR TS=(renal failure) OR TS=(renal insufficiency) OR TS=(liver disease) OR TS=(liver function) OR TS=(liver fibrosis)) AND (TS=(organophosphorus pesticide) OR TS=(OPs) OR TS=(organophosphorus insecticide))
1939

Ovid:
1 organphosphorus pesticide.mp. [mp=tx, bt, ti, ab, ct, sh, ot, nm, hw, fx, kf, ox, px, rx, an, ui ds, on, sy]
2 organophosphorus insecticide.mp. [mp=tx, bt, ti, ab, ct, sh, ot, nm, hw, fx, kf, ox, px, rx, an, ui ds, on, sy]
3 OPs.mp. [mp=tx, bt, ti, ab, ct, sh, ot, nm, hw, fx, kf, ox, px, rx, an, ui ds, on, sy]
4 cardiovascular disease.mp. [mp=tx, bt, ti, ab, ct, sh, ot, nm, hw, fx, kf, ox, px, rx, an, ui ds, on, sy]
5 heart disease.mp. [mp=tx, bt, ti, ab, ct, sh, ot, nm, hw, fx, kf, ox, px, rx, an, ui ds, on, sy]
6 diabetes mellitus.mp. [mp=tx, bt, ti, ab, ct, sh, ot, nm, hw, fx, kf, ox, px, rx, an, ui ds, on, sy]
7 T2DM.mp. [mp=tx, bt, ti, ab, ct, sh, ot, nm, hw, fx, kf, ox, px, rx, an, ui ds, on, sy]
8 diabetes.mp. [mp=tx, bt, ti, ab, ct, sh, ot, nm, hw, fx, kf, ox, px, rx, an, ui ds, on, sy]
9 respiratory disease.mp. [mp=tx, bt, ti, ab, ct, sh, ot, nm, hw, fx, kf, ox, px, rx, an, ui ds, on, sy]
10 pulmonary disease.mp. [mp=tx, bt, ti, ab, ct, sh, ot, nm, hw, fx, kf, ox, px, rx, an, ui ds, on, sy]
11 lung disease.mp. [mp=tx, bt, ti, ab, ct, sh, ot, nm, hw, fx, kf, ox, px, rx, an, ui ds, on, sy]
12 kidney disease.mp. [mp=tx, bt, ti, ab, ct, sh, ot, nm, hw, fx, kf, ox, px, rx, an, ui ds, on, sy]
13 kidney failure.mp. [mp=tx, bt, ti, ab, ct, sh, ot, nm, hw, fx, kf, ox, px, rx, an, ui ds, on, sy]
14 renal disease.mp. [mp=tx, bt, ti, ab, ct, sh, ot, nm, hw, fx, kf, ox, px, rx, an, ui ds, on, sy]
15 renal failure.mp. [mp=tx, bt, ti, ab, ct, sh, ot, nm, hw, fx, kf, ox, px, rx, an, ui ds, on, sy]
16 renal insufficiency.mp. [mp=tx, bt, ti, ab, ct, sh, ot, nm, hw, fx, kf, ox, px, rx, an, ui ds, on, sy]
17 liver disease.mp. [mp=tx, bt, ti, ab, ct, sh, ot, nm, hw, fx, kf, ox, px, rx, an, ui ds, on, sy]
18 liver function.mp. [mp=tx, bt, ti, ab, ct, sh, ot, nm, hw, fx, kf, ox, px, rx, an, ui ds, on, sy]
19 liver fibrosis.mp. [mp=tx, bt, ti, ab, ct, sh, ot, nm, hw, fx, kf, ox, px, rx, an, ui ds, on, sy]
12 4 or 5 or 6 or 7 or 8 or 9 or 10 or 11 or 12 or 13 or 14 or 15 or 16 or 17 or 18 or 19

13 1 or 2 or 3
14 12 and 13
1277

Embase: ('organophosphorus pesticide':ti,ab,kw OR 'organophosphorus insecticide':ti,ab,kw OR 'ops':ti,ab,kw) AND ('cardiovascular disease':ti,ab,kw OR 'heart disease':ti,ab,kw OR 'diabetes mellitus':ti,ab,kw OR 'DM':ti,ab,kw OR 'T2DM':ti,ab,kw OR 'diabetes':ti,ab,kw OR 'respiratory disease':ti,ab,kw OR 'pulmonary disease':ti,ab,kw OR 'lung disease':ti,ab,kw OR 'kidney disease':ti,ab,kw OR 'kidney failure':ti,ab,kw OR 'renal disease':ti,ab,kw OR 'renal failure':ti,ab,kw OR 'renal insufficiency':ti,ab,kw OR 'liver disease':ti,ab,kw OR 'liver function':ti,ab,kw OR 'liver fibrosis':ti,ab,kw)
225

Cochrane Library
ID Search Hits
#1 organophosphorus pesticide
#2 organophosphorus insecticide
#3 OPs
#4 diabetes mellitus
#5 DM
#6 T2DM
#7 diabetes
#8 respiratory disease
#9 pulmonary disease
#10 lung disease
#11 cardiocascular disease
#12 heart disease
#13 kidney disease
#14 kidney failure
#15 renal disease
#16 renal failure
#17 renal insufficiency
#18 liver disease
#19 liver function
#20 liver fibrosis
#21 (#1 or #2 or #3) AND (#4 or #5 or #6 or #7 or #8 or #9 or #10 or #11 or #12 or #13 or #14 or #15 or #16 or #17 or #18 or #19 or #20)
65
