# Supplementary material for: The Associations between Organophosphate Pesticides (OPs) and Respiratory Disease, Diabetes Mellitus, and Cardiovascular Disease: A Review and Meta-Analysis of Observational Studies
Source: Toxics. 2023 Aug 30;11(9):741. doi: 10.3390/toxics11090741 (PMC10535340; doi:10.3390/toxics11090741)
Supplement: Supplementary file 1 [file toxics-11-00741-s001.zip › Supplementary File S2.pdf]

Additional file 2

Figure of contents:

Figure	pages
**Figure S1.** Funnel plot for the association between organophosphate pesticides and respiratory diseases.	2
**Figure S2.** The significant association between individual organophosphate pesticides exposure and respiratory diseases.	3
**Figure S3.** The association between organophosphate pesticides exposure and participant's ethnicity (America and Asian) in respiratory diseases group.	4
**Figure S4.** The association between organophosphate pesticides exposure and participant's gender in respiratory diseases group.	5
**Figure S5.** The association between organophosphate pesticides exposure and study design in respiratory diseases group.	6
**Figure S6.** Funnel plot for the association between organophosphate pesticides and diabetes mellitus.	7
**Figure S7.** The association between organophosphate pesticides exposure and study design in diabetes mellitus group.	8
**Figure S8.** Funnel plot for the association between organophosphate pesticides and cardiovascular disease.	9

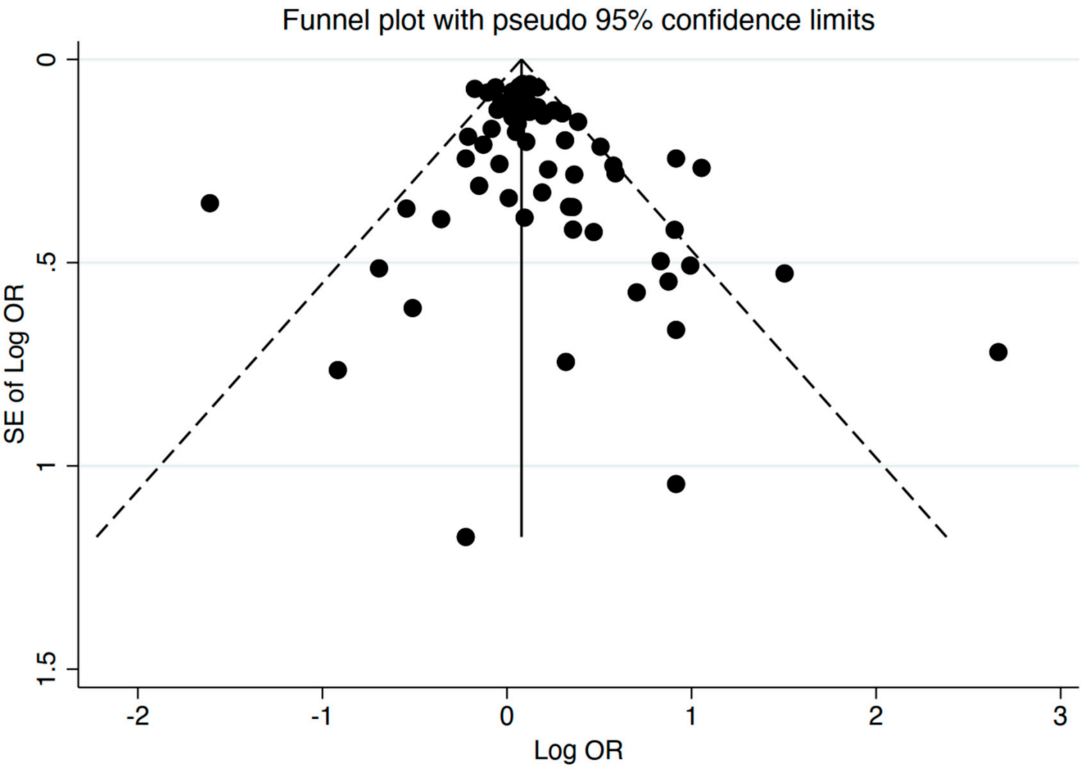

Figure S1. Funnel plot for the association between organophosphate pesticides and respiratory diseases.

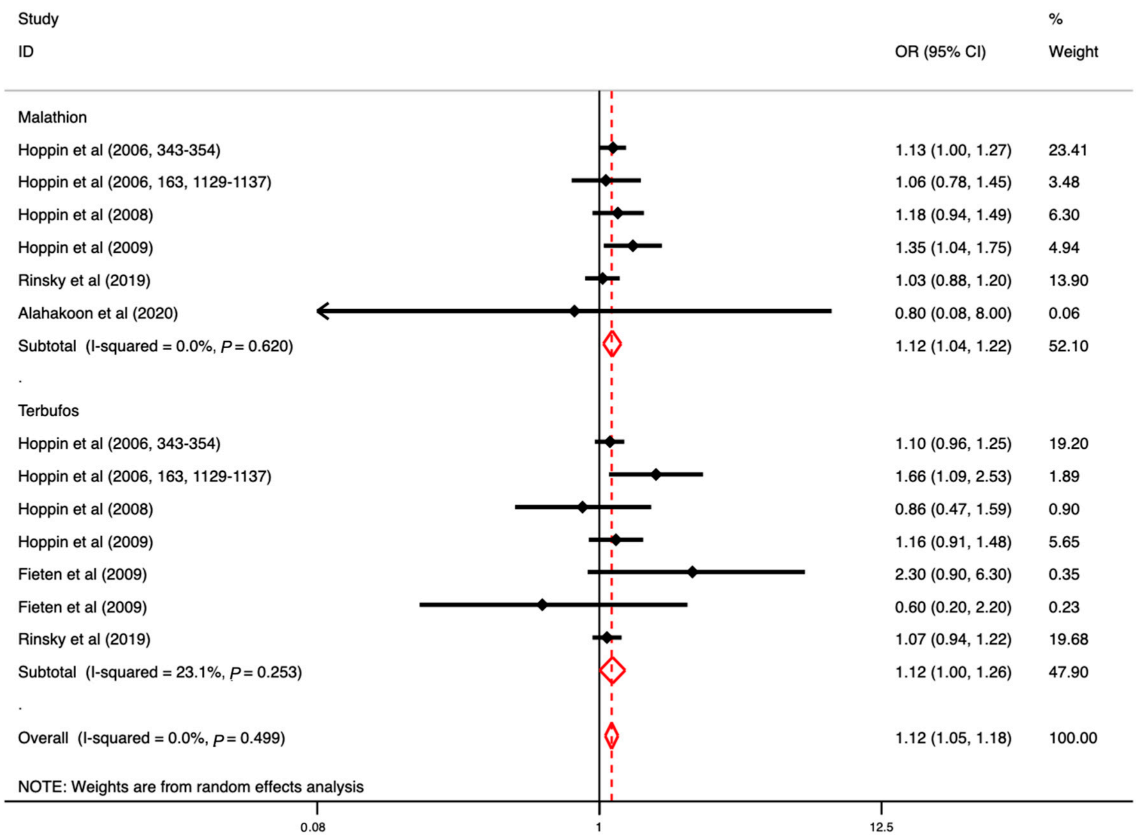

Figure S2. The significant association between individual organophosphate pesticides exposure and respiratory diseases.

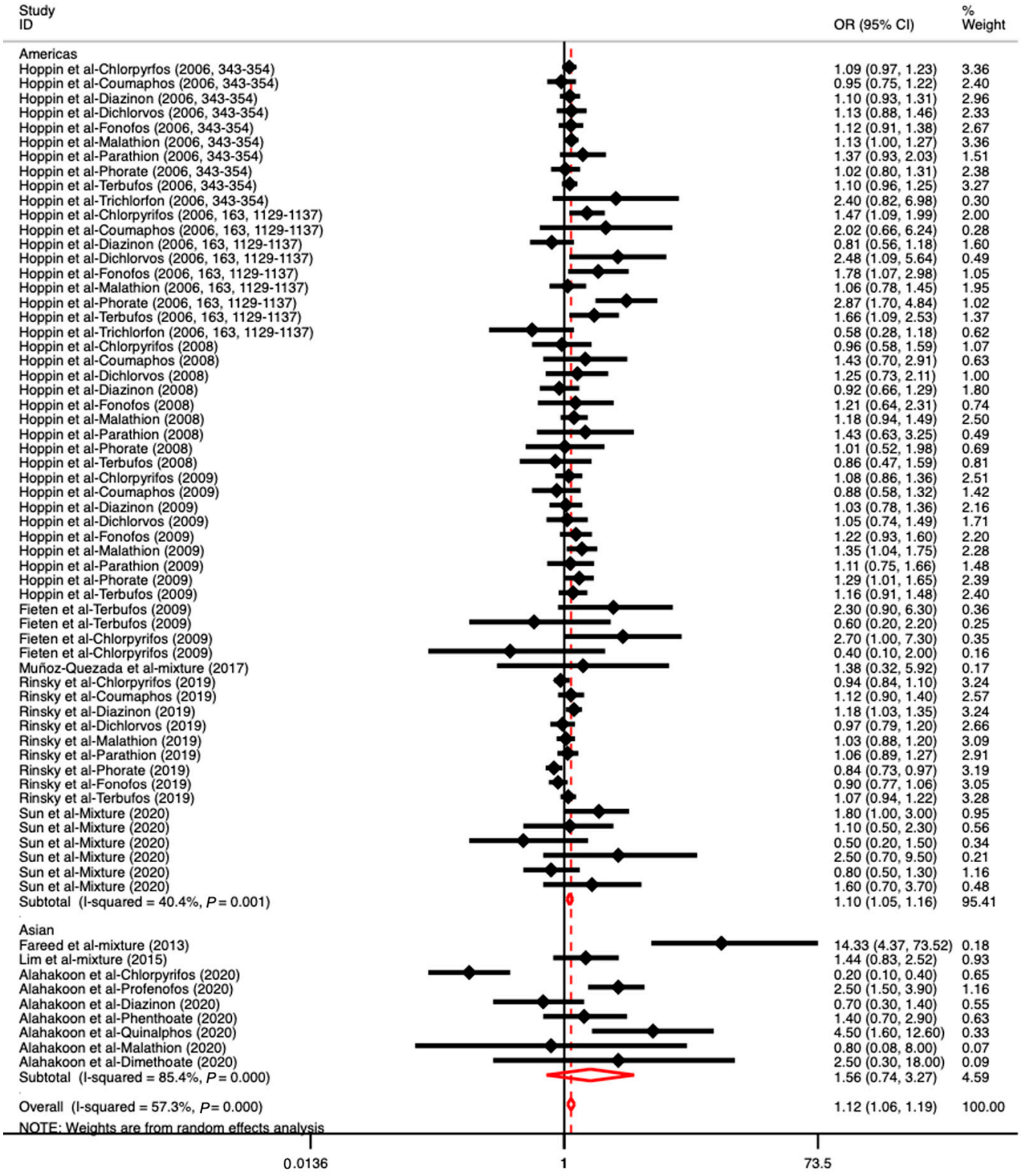

Figure S3. The association between organophosphate pesticides exposure and participant's ethnicity (America and Asian) in respiratory diseases group.

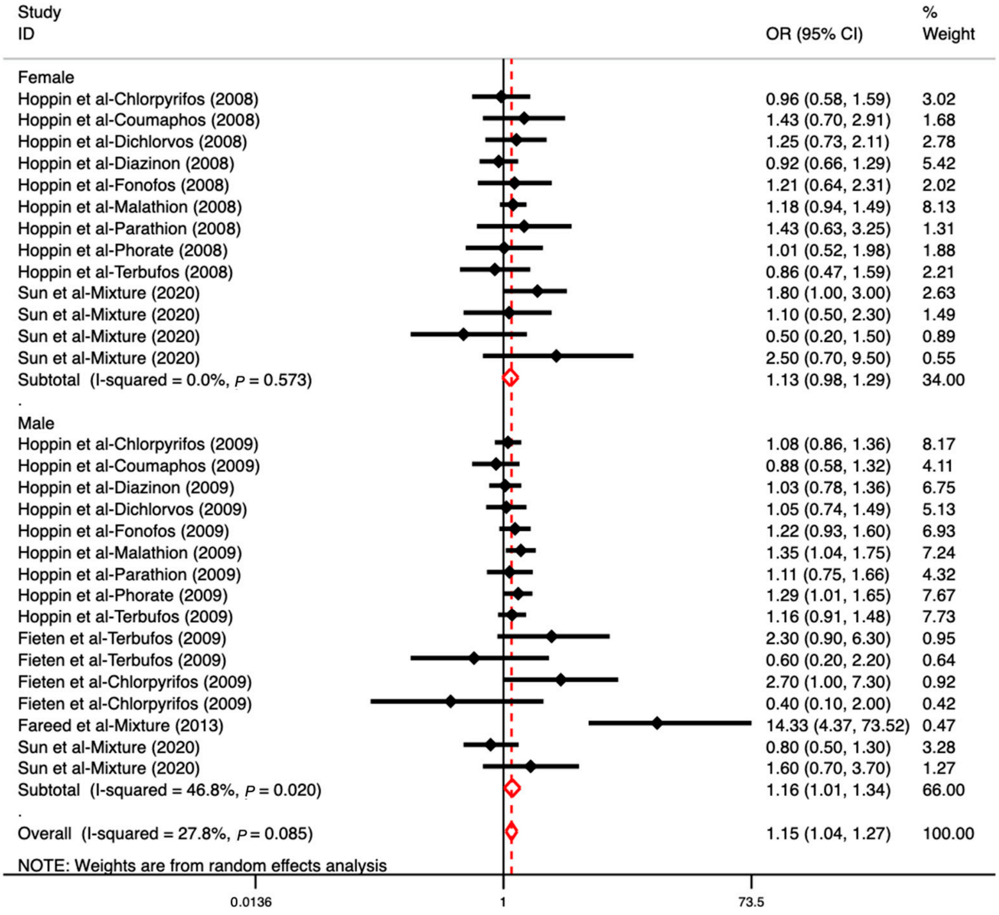

Figure S4. The association between organophosphate pesticides exposure and participant's gender in respiratory diseases group.

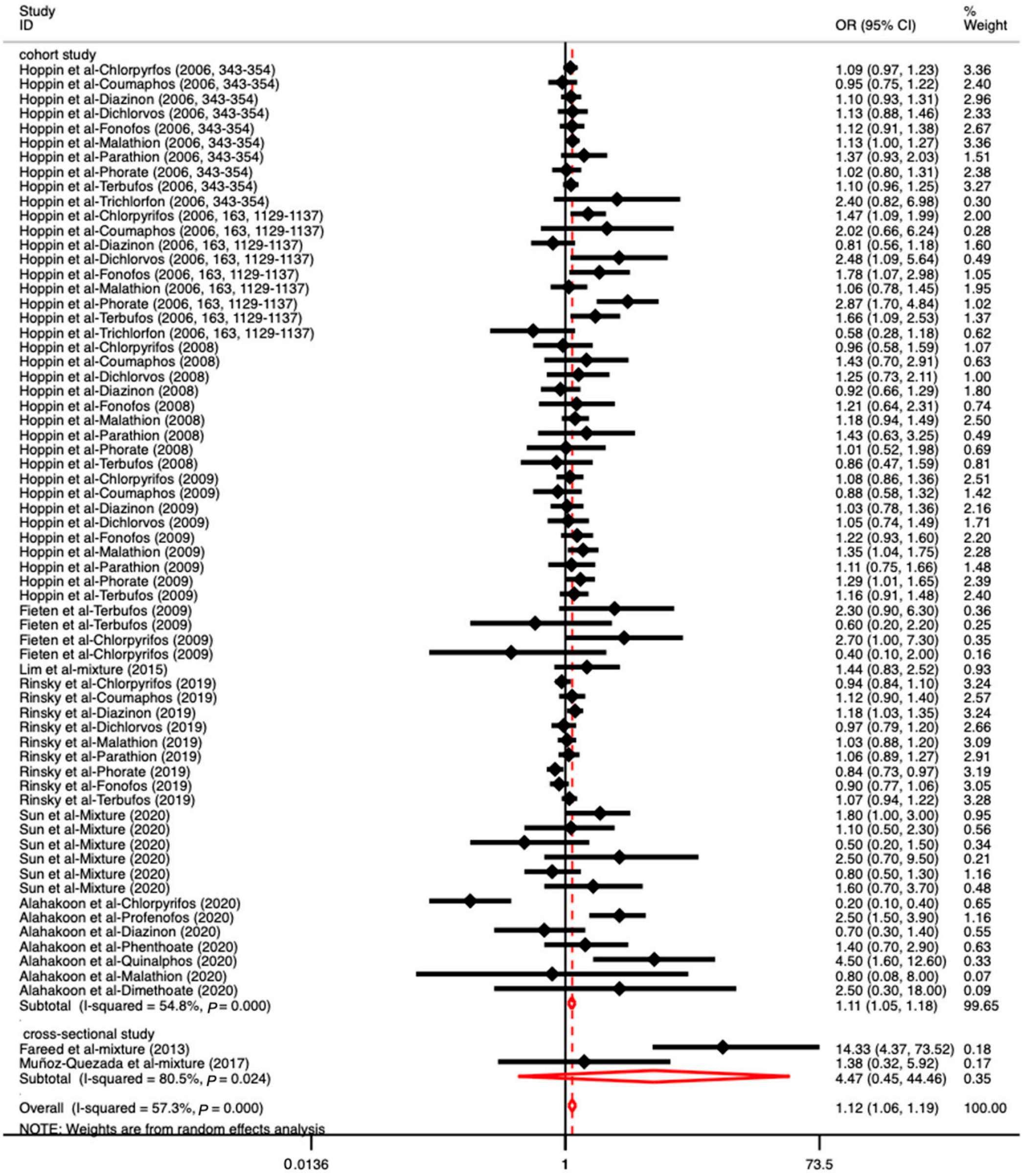

Figure S5. The association between organophosphate pesticides exposure and study design in respiratory diseases group.

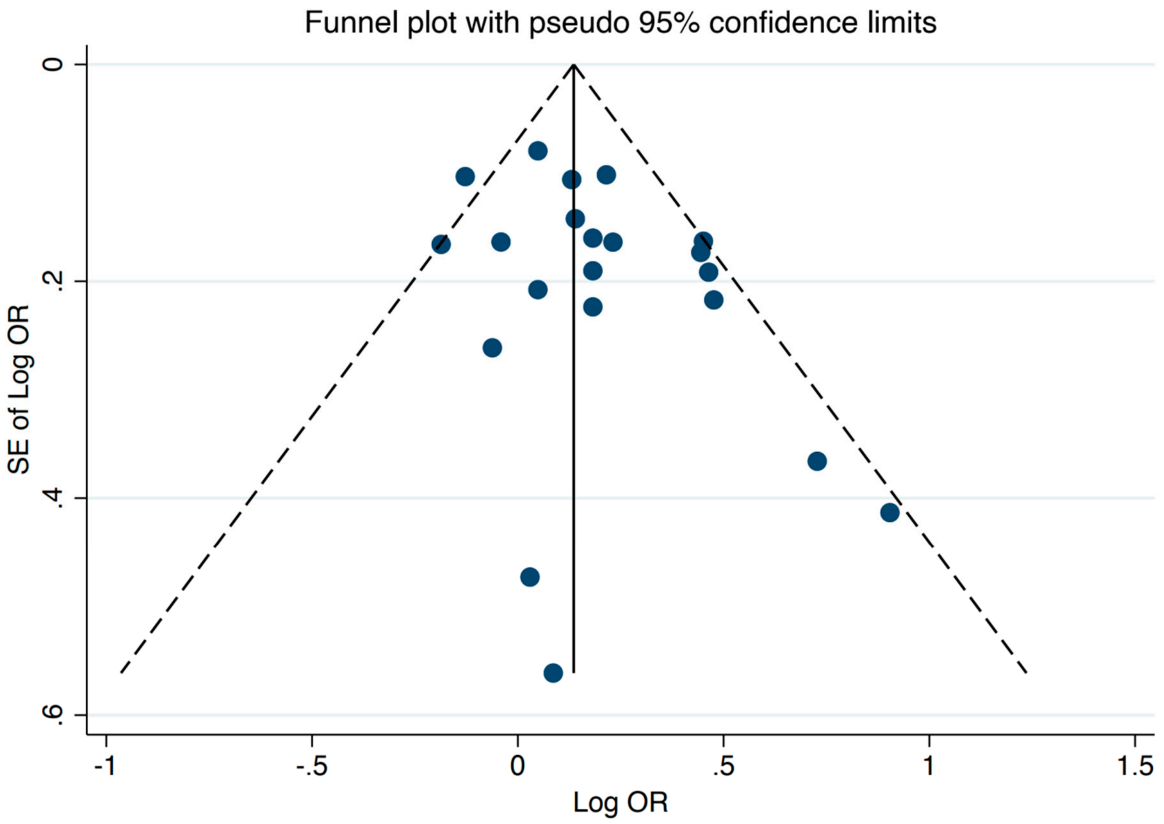

Figure S6. Funnel plot for the association between organophosphate pesticides and diabetes mellitus.

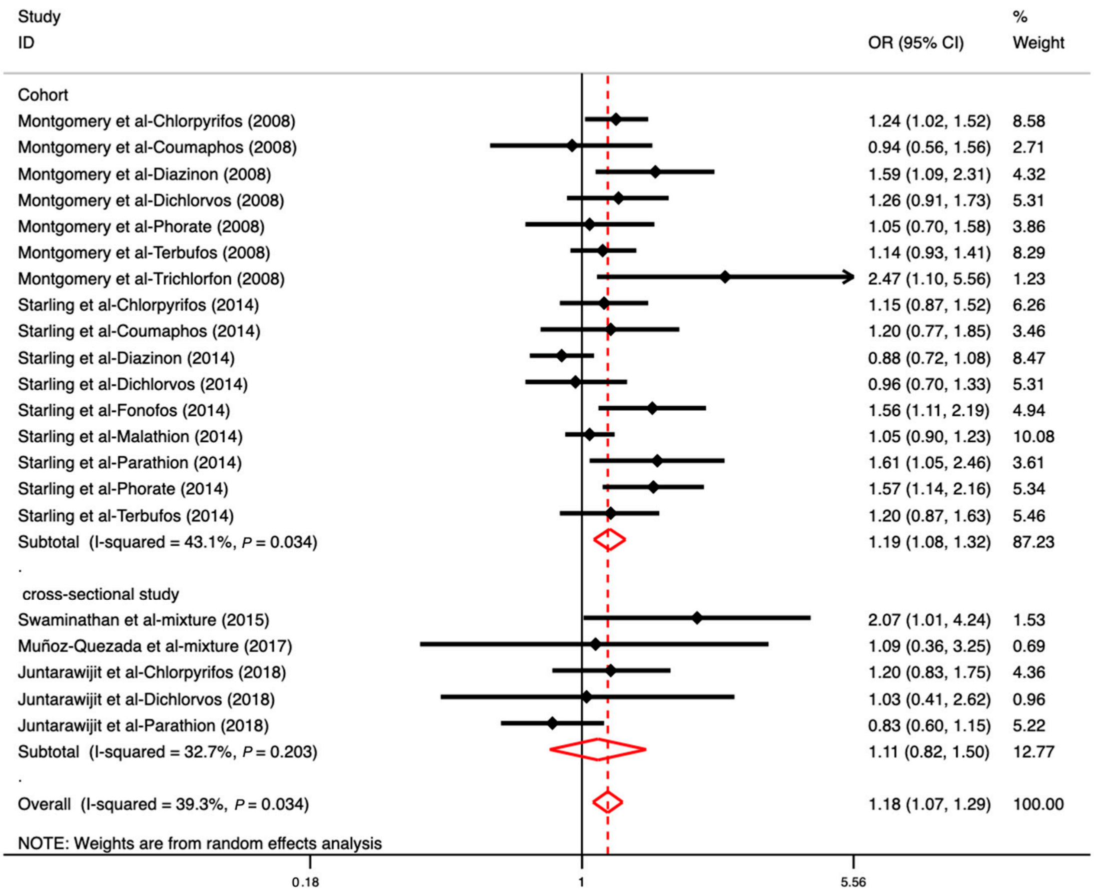

Figure S7. The association between organophosphate pesticides exposure and study design in diabetes mellitus group.

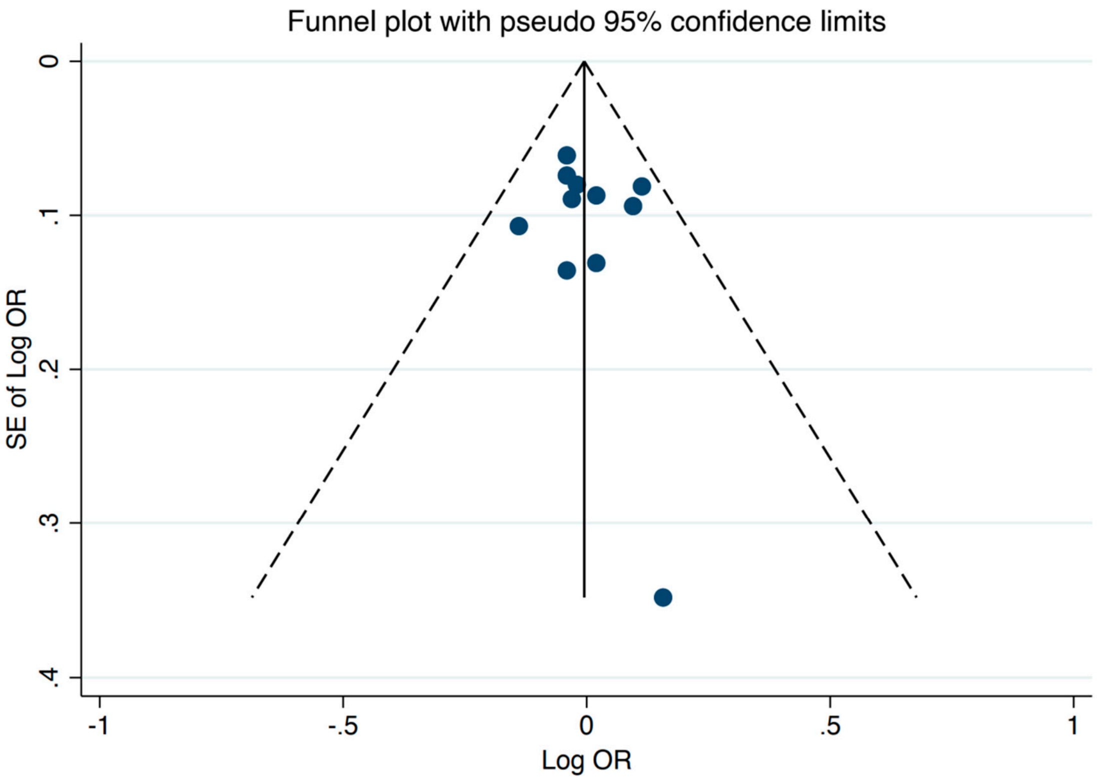

Figure S8. Funnel plot for the association between organophosphate pesticides and cardiovascular disease.
